# Systematic review: Do patient expectations influence treatment outcomes in total knee and total hip arthroplasty?

**DOI:** 10.1186/1477-7525-10-152

**Published:** 2012-12-18

**Authors:** Tsjitske M Haanstra, Tobias van den Berg, Raymond W Ostelo, Rudolf W Poolman, Ilse P Jansma, Pim Cuijpers, Henrica CW de Vet

**Affiliations:** 1 Department of Epidemiology and Biostatistics and the EMGO Institute for Health and Care Research, VU University Medical Centre Amsterdam, Van der Boechorststraat 7, 1081 BT, Amsterdam, the Netherlands; 2 Department of Health Sciences and the EMGO Institute for Health and Care Research, Faculty of Earth and Life Sciences, VU University Amsterdam, Amsterdam, the Netherlands; 3 Department of Orthopaedic Surgery and Department of Joint Research, Onze Lieve Vrouwe Gasthuis, Amsterdam, the Netherlands; 4 Medical Library, VU University Amsterdam, Amsterdam, the Netherlands; 5 Department of Clinical Psychology and the EMGO Institute for Health and Care Research, VU University Amsterdam, Amsterdam, the Netherlands

**Keywords:** Outcome expecations, Self efficacy expectations, Systematic review, Total knee arthroplasty, Total hip arthroplasty

## Abstract

**Objective:**

This systematic review aims to summarise all the available evidence related to the association between pre-operative patient expectations (outcome expectations, process expectations and self efficacy expectations) and 5 different treatment outcomes (overall improvement, pain, function, stiffness and satisfaction) in patients with total knee or total hip arthroplasty at three different follow-op periods (>6 weeks; >6 weeks- ≤6 months; >6 months).

**Methods:**

English and Dutch language articles were identified through PubMed, EMBASE.com, PsycINFO, CINAHL and The Cochrane Library from inception to September 2012. Articles assessing the association between pre-operative patient expectations and treatment outcomes for TKA/THA in either adjusted or unadjusted analysis were included. Two reviewers, working independently, determined eligibility, rated methodological quality and extracted data on study design, population, expectation measurements, outcome measurements and strength of the associations. Methodological quality was rated by the same reviewers on a 19 item scale. The scores on the quality assessment were taken into account when drawing final conclusions.

**Results:**

The search strategy generated 2252 unique references, 18 articles met inclusion criteria. Scores on the methodological quality assessment ranged between 6% and 79%. Great variety was seen in definitions and measurement methods of expectations. No significant associations were found between patient expectations and overall improvement, satisfaction and stiffness. Both significant positive and non-significant associations were found for the association between expectations and pain and function.

**Conclusions:**

There was no consistency in the association between patients’ pre-operative expectations and treatment outcomes for TKA and THA indentified in this systematic review. There exists a need for a sound theoretical framework underlying the construct of ‘patient expectations’ and consistent use of valid measurement instruments to measure that construct in order to facilitate future research synthesis.

## Background

Total hip and total knee arthroplasty (THA and TKA) are amongst the most cost-effective treatments within the field of orthopaedics [1,2]. Nevertheless the proportion of patients with unsuccessful outcomes is substantial ranging from 10-30% [3-10]. Even after technically well performed surgery patients may have residual complaints. In these cases post-operative imaging shows no abnormalities and low-grade infection or loosening is ruled out; but patients may still have impaired function and pain, resulting in a low quality of life and high health care costs [11]. It is therefore important for clinicians to know which factors, apart from technical factors, could possibly have an influence on the outcome after TKA or THA to improve their decision making and recommendations to patients opting for TKA or THA.

There is growing body of literature that suggests that patients’ expectations are associated with clinical outcomes in many different treatments, and therefore may be an important predictor for treatment outcomes. For example a strong predictive role of expectations was found in the systematic review by Iles et al. [12] who investigated recovery expectations in people with acute non specific low back pain. Constantino et al. [13] concluded in their systematic review that there was a small significant effect of outcome expectations on outcomes in psychotherapy.

Several studies have investigated the predictive capacity of expectations for outcomes of Total Knee and Total Hip Arthroplasty. However, inconsistencies are found in the results of these studies. Some studies seem to suggest that patient expectations explain a substantial part of the variance found in the outcomes of TKA or THA and draw strong conclusions and recommendations based on those findings [14,15]. However, other studies do not support these positive findings [16,17]. This inconsistency may be due to the different types of expectations measured, the different outcomes or the timing of the measures.

A systematic review could provide clarity as to which types of expectations are associated with which outcomes at which time points and thereby may inform researchers and orthopaedic clinicians in their usage of patients’ pre-operative expectations as a possible predictor of outcomes.

In the current study we therefore aim to summarise all the available evidence for the association between three types of expectations namely; outcome expectations, self-efficacy expectations and process expectations, and five common types of treatment outcomes within TKA/THA research namely; overall improvement, pain, function, stiffness and satisfaction at three different follow-up periods namely; ≤6 weeks, 6 weeks- ≤6 months and >6 months.

## Methods

### Definitions used in this review

Recent literature makes a distinction between expectancies and expectations, though in earlier research, these terms are used interchangeably. For the purposes of this review we defined expectancy as “the act or state of expecting” and expectations as “cognitions regarding probable future events”. As we will review the literature about the association between patients’ cognitions regarding their TKA or THA and the outcomes of TKA or THA, this review will solely focus on expectations.

We defined expectations by adopting three key concepts from Bandura’s self-efficacy theory [18,19] and an extensive literature review on the role of expectancies in the placebo effect by Crow et al. [20].

• Outcome expectations: beliefs that certain actions will achieve particular outcomes.

• Process expectations: beliefs about the content and process of interventions.

• Self-efficacy expectations: beliefs in one’s capabilities to organize and execute the courses of action required to produce given attainments.

### Data sources and searches

A comprehensive systematic search was done by TMH and EPJ in the bibliographic databases PubMed, EMBASE.com, PsycINFO (via CSA Illumina), Cinahl (via EBSCO) and The Cochrane Library (via Wiley) from inception to September 20^th^ 2012.

Search terms included controlled terms from MeSH in PubMed, EMtree in EMBASE.com, thesaurus terms in PsycINFO and Subject Headings in Cinahl as well as free text terms. We used free text terms only in The Cochrane library. Search terms expressing ‘total hip and total knee arthroplasty’ were used in combination with Boolean AND search terms comprising ‘expectations, self efficacy and health knowledge’. The full search strategy for PubMed can be found in Additional file 1. We adapted the PubMed search strategy for the other databases, the full search strategies for these databases are available upon request. Furthermore, the references of the identified articles were searched for relevant publications.

### Study selection

The studies had to meet the following inclusion criteria:

• The patients were adults (>18 years, no upper limit) who received total hip or total knee arthroplasty.

The design of the study had to be a prospective longitudinal cohort. Retrospective studies were not included due to the potential bias in this type of study.

• Expectations had to be measured before surgery.

• The association between pre-operative expectations and one or more out of 5 different treatment outcomes namely overall improvement, pain, function, stiffness and satisfaction had to be tested either in a unadjusted or adjusted analysis.

• The article had to be written in English or Dutch.

Studies assessing the association between whether expectations were met after surgery and treatment outcomes were excluded.

Two reviewers (TMH and TB) independently applied the inclusion criteria to the titles and abstracts. If it was not clear whether the studies met the inclusion criteria, the full text article was examined.

### Data extraction and methodological quality assessment

The same reviewers independently extracted the data using a data extraction form that included information on study design, population, the expectation measurements and the outcome measurements. Moreover, the strength of associations (i.e. correlation coefficients, p-values, odds ratio’s and regression coefficients) stating the correlation between expectations and outcomes were extracted. Moreover the reviewers independently scored the methodological quality of the selected studies on the basis of the following 19 item scale which was adapted from the methodological quality assessment for observational studies developed by Hayden et al. [21].

### Study participation

1. Is the source population adequately described? (primarily in terms of indication for the operation).

2. Is it clear how participants are recruited? (consecutive, random or selective sample).

3. Are in and exclusion criteria described?

4. Is the chance of selection bias small? (is the study population an adequate representation of the source population).

5. Are at least five out of six key baseline characteristics of the study population reported? (gender, age, type of operation, indication for THA/TKA, baseline pain and function, co-morbidities).

### Measurement of determinant

6. Is there a clear definition or description of the type of expectations measured? (categories: outcome expectations, self efficacy, process expectations).

7. Is it clear how expectations are measured? (questionnaire/interview, number of items, continuous/ordinal/dichotomous).

8. Does an adequate proportion of the (eligible) study sample have complete data for the expectation measurement? (>80% is adequate).

### Outcome measurement

9. Is a clear definition of the outcome of interest provided?

10. Is it clear **how** the outcome is measured? (questionnaire/interview/functional assessment, number of items, continuous/ordinal/dichotomous).

11. Is the response rate for the outcome adequate? (>80% is adequate).

12. Is it plausible that there is no selective drop-out during follow up?

13. If there is missing data, are they dealt with in the appropriate way?

14. Is the outcome measure blinded for exposure status?

### Confounding measurement and account

15. Are at least three out of four important categories of confounders measured? (patient characteristics, surgery characteristics, baseline disease characteristics, psychosocial characteristics).

16. Are appropriate methods used to account for the confounders in the analyses?

### Analysis

17. Is an appropriate statistical method used for the analyses?

18. Are continuous variables (determinant or outcome) not dichotomized in the analyses?

19. Are the number of observations in the final multivariable model at least 10 times the number of independent variables in the analysis?

Each criterion was answered using ‘yes’ (criterion fulfilled), ‘no’ (criterion not fulfilled) or ‘question mark’ (unclear whether criterion is fulfilled). The sum of all the positively scored items, divided by the number of relevant items for each study provided us with a total score for each study (the higher the score, the higher the methodological quality). The scores on this quality assessment were taken into account when drawing conclusions about the strength of evidence.

For the selection of studies, the data extraction and the quality assessment a consensus strategy was used to resolve the disagreements between the two reviewers, if consensus could not be reached, a third reviewer (RWO) was consulted.

### Data syntheses and analysis

In the study protocol a meta-analysis was planned for each of the five outcomes. However, due to the heterogeneity in studies, especially in how patients’ expectations were measured, it was not possible to statistically pool the results of the individual studies. Therefore, we only qualitatively summarize the evidence (in terms of the direction and strength of the associations, the sample size and methodological quality).

The p-values presented in the original articles were regarded statistically significant when they were smaller than 0.05. A correlation coefficient above 0.3 and below −0.3 was regarded of significant importance [22]. In the results table a ‘+’ corresponds with a positive correlation that implies that high expectations are associated with a better outcome (i.e. less pain or better function). A ‘–‘ corresponds with a negative correlation and implies that higher expectations are associated with a worse outcome. A ‘+−‘ corresponds with an unclear association (e.g. both positive and negative associations are observed). A ‘0’ represents that there is no association found.

In a number of studies multiple expectation measurements were used and therefore multiple associations were reported regarding the same outcome, for instance, outcome expectations were measured as 1. the expectation for post-operative pain and 2. the expectation for post-operative functioning. In those cases we used the expectation measure that conceptually is closest related to the outcome in our analysis for example when the outcome was ‘pain’, the association between expectations about post-operative pain and post-operative pain was regarded the association of interest and not the association between expectations about post-operative functioning and post-operative pain. When we refer to validated measurement instruments we mean that these instrument have shown to have sufficient clinimetric properties (content and external validity) for the TKA/THA population [23].

## Results

**Figure 1:**
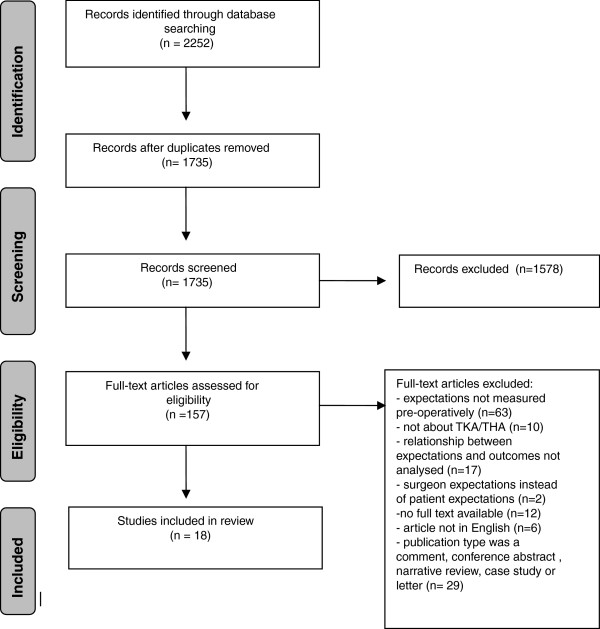
Flowchart of literature search and selection process.

The literature search generated a total of 2252 references: 528 in PubMed, 1091 in EMBASE.com, 33 in PsycINFO, 554 in Cinahl and 46 in The Cochrane Library. After removing duplicates (n= 517) 1735 papers remained. After assessing the titles and abstracts 157 full text articles were retrieved for further investigation. A total of 18 articles met all inclusion criteria and were included in this review (Figure 1).

### Study characteristics

Table 1 shows selected characteristics of the included studies.

**Table 1:** Characteristics of the included studies

**Study**	**Quality score (%)**	**N**	**Hip or knee**	**Type of expectations**	**Measure of expectations**	**Timing of expectation measurement**	**Health outcome measures**	**Timing of outcome measures**
**Vd Akker-Scheek 2007 **[30]	79	123	Knee	Self efficacy expectations	- Self efficacy for rehabilitation outcome scale	6 weeks prior to surgery	1. WOMAC overall score	6 months post surgery
							2. SF-36 physical function	
**Arden 2011**[35]	50	799	Hip	Outcome expectations	2 items	Pre-operative	1. Satisfaction	1 year and 2 years post surgery
					- How painful do you expect your hip to be when you are fully recovered from surgery (not at all painful, slightly painful, very painful)	No specific time point reported	2. Change in oxford hip score	
					- How limited do you expect to be in your usual activities when you are fully recovered from surgery (not limited at all, slightly limited, moderately limited, greatly limited)			
**Brokelman 2008**[24]	50	44	Knee and hip	Outcome expectations	- VAS (0–100) for expectation of satisfaction with surgery 1 year postoperative	After pre-operative information meeting	1. VAS for Satisfaction	Mean = 13.1 months post surgery
**Cross 2009**[16]	39	106	Knee and hip	Outcome expectations	- Hospital for Special Surgery Hip or Knee Replacement Expectations Questionnaire	Pre-operative	1. WOMAC pain change	6 months post surgery
				Self efficacy expectations	- Arthritis Self Efficacy Scale	No specific time point reported	2. WOMAC function change	
							3. WOMAC stiffness change	
**Engel 2004**[25]	58	78	Knee	Outcome expectations	- Expected probability of recovery (VAS)	2 weeks prior to surgery	1. WOMAC pain	6 months post surgery
				Self efficacy expectations	- Expected change in quality of life (4 response choices)		2. WOMAC function	
					- Coping efficacy (two items, 5 point Likert-type scaled)		3. WOMAC stiffness	
							4. Overall recovery (clinician assessment)	
**Gandhi 2009**[14]	58	2350	Knee and hip	Outcome expectations	3 ‘open’ questions	Pre-operative	1. WOMAC pain change	1 year post surgery
					- Time to fully recover from surgery	No specific time point reported	2. WOMAC function change	
					- Pain expected after surgery			
					- Ability to perform usual activities when recovered			
**Hartley 2008**[36]	53	100	Knee and hip	Self efficacy expectations	- Self efficacy for rehabilitation outcome scale	1 month prior to surgery	1. Harris Hip Scale / The American Knee Society Score (function)	6 weeks post surgery
					- 12 items about outcome expectations with Likert type scales from 0–10.			
**Judge 2011**[34]	63	1327	Hip	Outcome expectations	Number of expectations answered on the following open answered item:	Pre-operative	1. Improved/not improved according to the OMERACT-	1 year post surgery
					- What things do you think your might be able to do in a year’s time, that you NEED to be able to do, but CANNOT do now, if the operation is a total succes	No specific time point reported	OARSI criteria	
							2. WOMAC pain	
							3. WOMAC stiffness	
							4. WOMAC function	
**Lingard 2006**[26]	53	598	Knee	Outcome expectations	4 items answered on a 5 point Likert-type scale (not at all important – very important).	6 weeks prior to surgery		1 year post surgery
					- Expectations regarding pain level		1. WOMAC pain	
					- Expectations regarding walking distance		2. WOMAC function	
					- Expectations regarding limitation of recreational activity		3. Satisfaction	
					- Expectations regarding the use of a walking aid			
**Lopez-Olivo 2011**[32]	74	272	Knee	Self efficacy expectations	Arthritis Self Efficacy Scale	Within a month prior to surgery	1. WOMAC pain	6 months post surgery
							2. WOMAC function	
							3. KSRS total	
							4. KSRS knee score	
							5. KSRS function	
**Mahomed 2002**[15]	58	222	Knee and hip	Outcome expectations	2 items answered on a 4 point Likert type scale	Prior to surgery	1. WOMAC pain	6 months post surgery
					- Limitations in activities of daily living	No specific time point stated	2. WOMAC function	
					- Pain relief		3. WOMAC stiffness	
					2 items answered on a VAS 0-100			
					- Overall success			
					- Likelihood of complications			
**Mannion 2009**[27]	58	146	Knee	Outcome expectations	- Expected time to full recovery (open answer in months)	Approximately 2 weeks prior to surgery	1. Global outcome/result of surgery	2 years post surgery
					- Expected pain after recovery from surgery (not at all through very painfull)		2. Satisfaction with surgery	
					- Expected limitations in everyday activities after recovery from surgery (not at all limited through greatly limited)			
**Nilsdotter 2009**[28]	44	102	Knee	Outcome expectations	-Expectations in relation to walking ability measured on a 6 point likert type scale	Pre-operative, no exact time point stated	1. SF-36 physical function	6 months,
								1 year and
								5 years post surgery
					-Expectations in relation to leisure activities measured on a 7 point likert scale			
					-Estimated time to recovery. And also about the 5 KOOS domains			
					-Expectations dichotomized in high and low expectations			
**Oettingen 2002**[33]	68	51	Hip	Outcome expectations	5 items answered on 5 point Likert type scales combined in a single score.	On the day of admission to the hospital	1. Range of hip motion (3 items, rated by PT)	Two weeks post surgery
					-How likely do you think it is that two weeks after surgery you will be able to go for a brief walk using an assistive cane		2. Walking on stairs (1 item rated by PT),	
					-How likely do you think it is that two weeks after surgery you will be walking on stairs up and down with the help of an assistive cane?		3. Recovery. How well the patient does in comparison to other patients (4 items, rated by PT)	
					-How functionally able do you think you will be 3 months after surgery		Al questions were answered on a 5 point likert type scale.	
					-To what extent do you think you will be without pain 3 months after surgery			
								
**Quintana 2009**[17]	74	788	Hip	Outcome expectations	1 item about expectation of pain relief	Whilst patients were on the waiting list	1. WOMAC pain	6 months and 2 years post surgery
							2. WOMAC function	
							3. WOMAC stiffness	
**Riddle 2009**[29]	68	157	Knee	Self efficacy expectations	Arthritis self efficacy scale short form	Preoperatively, no specific time point mentioned	1. WOMAC pain	6 months post surgery
							2. WOMAC function	
**Suda 2010**[37]	6	130	Knee and hip	Outcome expectations	Expectations in relation to 5 items:	1 month pre-operative	1. Harris Hip scores	3 years post surgery
					-Pain			
					-Change in personal relationships			
					-Decrease in worries about life			
					-Resuming old hobbies			
					-Overall expectations of the new joint			
					-The FFbH-OA-survey adapted to expectations			
**Vissers 2010**[31]	61	45	Knee	Outcome expectations	2 items answered on a 4 point Likert type scale	+− 6 weeks pre-operative	1. Satisfaction with surgery	6 months post surgery
					-Limitations in activities of daily living			
					-Pain relief			
					1 item answered on a VAS 0-100			
					-Overall success			

WOMAC= Western Ontario and McMaster Universities Arthritis Index, VAS= Visual Analogue Scale, PT=Physiotherapist, KOOS=Knee injury and Osteoarthritis Outcome Score, FFbH-OA= Functional Questionnaire of Hannover for Osteoarthritis, KSRS= Knee Society Rating System.

The number of participants in the included studies ranged from 44 [24] to 2350 [14]. Eight studies only included knee arthroplasties [25-32], four studies only included hip arthroplasties [17,33-35] and the remaining six studies included both [14-16,24,36,37] of which four studies [14,15,24,36] presented their results for TKA and THA together and two [16,37] presented their results seperately. Thirteen studies only included primary arthroplasties, one [36] included primary as well as revision arthroplasties and four studies [25,34,35,37] did not report whether they included revision arthroplasties.

Studies included in this review defined patient expectations in many different ways, however, nine studies [14,16,17,24,29,31,34,35,37] did not provide a definition. From the definitions stated in the studies or the items of the expectation measurement we were able to classify the studies as either assessing outcome expectations, self efficacy expectations or process expectations. Four studies measured only self efficacy expectations [29,30,32,36], twelve studies solely addressed outcome expectations. Two studies measured both self efficacy and outcome expectations [16,25] and none measured process expectations. Five studies used validated questionnaires for their measurement of expectations [16,29,30,32,36]. Fifteen studies used a multiple item instrument to assess patient expectations ranging from 3 [14] to 19 [30] items and two studies [17,24] used a 1 item measurement, one study used an open ended item to elicit patients’ expectations, in this study the number of expectations stated was used in the analysis [34]. In all studies patient expectations were measured before surgery. Fifteen out of eighteen studies measured two or more outcomes [14-17,25-30,32-35,37]. Function and pain were the most common outcomes and were measured with validated instruments. Short term outcomes (≤6 weeks) were measured in two studies [33,36]. Nine studies [15-17,25,28-32] included at least one outcome measurement between 6 weeks and 6 months after surgery. Long term outcomes (>6 months) were measured in eight studies [14,24,26-28,34,35,37].

### Methodological quality

Studies scored between 6% [37] and 79% [30] of the maximum score on the quality assessment, with an average of 56%. In Table 2 scores on all items can be found. Best scores were derived on the items ‘Are inclusion criteria and exclusion criteria described?’, ‘Is a clear definition of the outcome of interest provided?’ and ‘Is it clear how the outcome is measured?’. As expected, the lowest score was for the item ‘is the outcome measure blinded for exposure status’ because most studies used self-reported questionnaires.

**Table 2:** Scores on the methodological quality assessment

	**1**	**2**	**3**	**4**	**5**	**6**	**7**	**8**	**9**	**10**	**11**	**12**	**13**	**14**	**15**	**16**	**17**	**18**	**19**	**Total score**	**%**
**Vd Akker- Scheek**	?	+	+	-	+	+	+	+	+	+	+	-	?	+	+	+	+	+	+	15/19	79
**Arden**	+	+	+	?	-	+	+	+	+	+	-	+	-	-	-	-	-	-	n/a	9/18	50
**Brokelman**	-	+	+	?	+	+	+	-	+	+	?	?	?	-	+	-	-	+	n/a	9/18	50
**Cross**	-	+	-	-	-	+	+	-	+	+	-	?	-	-	+	-	-	+	n/a	7/18	39
**Engel**	+	-	-	?	-	+	+	+	+	+	+	+	?	-	-	+	-	+	+	11/19	58
**Gandhi**	+	-	+	+	+	-	+	-	+	+	-	?	-	-	-	+	+	-	+	11/19	58
**Hartley**	-	-	+	?	-	+	+	-	+	+	-	-	-	-	+	+	+	+	+	10/19	53
**Judge**	+	-	+	?	+	+	+	?	+	+	-	-	+	-	+	+	+	-	+	12/19	63
**Lingard**	+	+	+	?	+	-	+	?	+	+	+	+	-	-	-	?	+	?	?	10/19	53
**Lopez Olivo**	+	+	+	?	+	+	+	?	+	+	+	?	-	-	+	+	+	+	+	14/19	74
**Mahomed**	+	?	+	-	+	-	+	-	+	+	+	?	-	-	+	+	+	-	+	11/19	58
**Mannion**	+	+	+	-	+	-	+	-	-	+	-	-	-	-	+	+	+	+	+	11/19	58
**Nilsdotter**	+	+	+	?	+	-	+	?	+	+	+	-	-	-	-	-	-	-	n/a	8/18	44
**Oettingen**	-	+	+	?	-	+	-	+	+	+	+	+	+	-	-	+	+	+	+	13/19	68
**Quintana**	+	+	+	+	+	-	-	+	+	+	-	+	-	-	+	+	+	+	+	14/19	74
**Riddle**	-	+	+	+	+	+	+	+	+	-	+	?	-	-	+	+	+	-	-	13/19	68
**Suda**	-	-	+	?	-	-	-	?	-	-	-	-	-	-	-	-	-	?	n/a	1/18	6
**Vissers**	+	?	+	+	+	+	+	?	+	+	+	+	+	-	+	-	-	-	n/a	11/18	61

### Associations between patient expectations and treatment outcomes

In Table 3 unadjusted associations (i.e. correlation coefficients, unadjusted beta coefficients or p-values for other tests) and adjusted associations are shown. Results are stratified by timing of outcome measure (≤6 weeks, >6 weeks - ≤6 months, >6 months) and type of expectation (outcome expectations and self efficacy expectations). The methodological quality score of the studies included in each association is reported as well as the number of participants.

**Table 3:** Unadjusted and adjusted associations between expectations and 5 treatment outcomes

	**Overall improvement**					**Pain**						**Function**				**Stiffness**					**Satisfaction**				
	**E1/E2**	**Association individual studies**	**Quality score**	**N**	**Overall association**	**E1/E2**	**Association individual studies**	**Quality score**	**N**	**Overall association**	**E1/E2**	**Association individual studies**	**Quality score**	**N**	**Overall association**	**E1/E2**	**Association individual studies**	**Quality score**	**N**	**Overall association**	**E1/E2**	**Association individual studies**	**Quality score**	**N**	**Overall association**
**Unadjusted associations**																									
≤ 6 weeks						E2	r=−0.08 [36]	53%	100	O	E2	r=−0.05 [36]	53%	100	O	E1	r=0.165 ‡ [16]^hips^	39%	106	0	E1	OR=1.176 (ns)∫ [31]	61%	45	O
>6 weeks – ≤6 months	E2	r=0.13 [30]	79%	123	O	E1	r=0.118* [16]^hips^	39%	106	+−	E1	r=0.170^†^[16]^hips^	39%	106	+−		r=−0.024 ‡ [16]^knees^	39%	106			OR=0.593 (ns)# [31]			
			r=0.24 [32]	74%	272			r=0.188* [16]^knees^	39%	106			r=−0.208^†^[16]^knees^	39%	106			ns [15]	58%	222		OR=1.000 (ns)∞ [31]			
							p=0.001 [14]	58%	2350			p<0.001 [14]	58%	2350			p=0.46 [17]	74%	788						
							p<0.05 [15]	58%	222			ns [15]	58%	222											
							p=0.62 [17]	74%	788			p=0.90 [17]	74%	788											
						E2	r=−0.12 [32]	74%	272		E2	r=0.19 [30]	79%	123	O										
												r=−0.19 [32]	74%	272											
> 6 months	E1	r=0.24 [27]	58%	146	+−	E1	p=0.012 [14]	58%	2350	+	E1	r=−0.547 [37]	6%	1302	+−	E1	OR= 1.23 (p<0.001) [34]	63%	1327	+	E1	r=−0.03 [24]	50%	44	+−
		p=0.70 ∏ [35]	50%	799			OR=1.17 (p<0.001) [34]	63%	1327			p<0.001 [14]	58%	350								r=0.274 ∫[27]	58%	146	
		p=0.013 Ω [35]	50%	799								p<0.001 [28]	44%	102								r=0.262^**#**^[27]	58%	146	
		OR=1.36 (=0.013) [34]	63%	1327								OR=1.25 (p<0.001) [34]	63%	1327								r=0.102§ [27]	58%	146	
**Adjusted associations**																						p=0.171 ∫ [35]	50%	799	
≤ 6 weeks	E1	β0.23 (p0.10) [33]	68%	51	O						E1	β 0.23 (p=0.11) [33]	68%	51	O							p=0.013 # [35]	50%	799	
											E2	β 0.151 (ns) [36]	53%	100	O										
>6 weeks – ≤6 months	E1	β-0.107(ns) [25]	58%	78	O	E1	β0.364 (p<.01)* [25]	58%	78	+−	E1	β 0.081 (ns) [25]	58%	78	+−	E1	ns [15]	58%	222	O					
							β7.8 (p<.01) [15]	58%	222			ns [15]	58%	222			ns [17]	74%	788						
							ns [17]	74%	788			β 8.10 (p<0.01)∞ [15]	58%	222											
	E2	β0.09 (ns) [30]	79%	123	0							ns § [17]	74%	788											
		β-0.193 (ns) [25]		78		E2	β-0.388 (p<.01)^†^[25]	58%	78	+−	E2	β0.18 [30]	78%	123	+−										
		ns [32]	58%	272			OR=0.80§ [29]	68%	157			β-0.337 (p<0.05) [25]	58%	78											
			74%					ns [32]	74%	272			ns § [29]	68%	157											
												ns [32]	74%	272												
> 6 months	E1	ns [27]	58%	146	+−	E1	p<0.001‡ [14]	58%	2350	+−	E1	p>0.05‡ [14]	58%	2350	+−	E1	OR= 1.21 (p=0.003) [34]	63%	1327	+	E1	ns [26]	53%	598	O	
		OR=1.34 (p = 0.04) [34]	63%	1327			p<0.05 [26]	52%	598			ns [26]	52%	598								ns ∫ [27]	58%	146		
							ns [17]	74%	788			ns [17]	74%	788								ns ^#^[27]	58%	146		
							OR=1.14 (p=0.049) [34]	63%	1327			OR=1.20 (p<0.001) [34]	63%	1327								ns § [27]	58%	146		

E1 outcome expectations, E2 efficacy expectations, ns= non significant, + positive association between expectations and outcome (the higher the expectations the better the outcome)**,** - negative association between expectations and outcome (the higher the expectations the worse the outcome)**,** O no association between expectations and outcome, +− unclear association between expectations and outcomes, ∏ association between expectations about pain and overall improvement, Ω association between expectations about function and overall improvement *correlation between importance of expectations and WOMAC pain change score between baseline and 6 months after surgery, †correlation between importance of expectations and WOMAC function change score between baseline and 6 months after surgery, **‡**correlation between importance of expectations and WOMAC stiffness change score between baseline and 6 months after surgery, ∫ association between expectations about pain and satisfaction, # association between expectations about functional limitations and satisfaction, § association between expectations about recovery time and satisfaction, ∞ association between expectations about overall success and satisfaction.

#### Overall improvement

Seven studies [25,27,30,32-35] included a measure of overall improvement. However, the method of measuring both the determinant (outcome expectations or self efficacy expectations) and the outcome (overall improvement) differed amongst the studies. In the studies by Engel et al. [25], Oettingen [33] and Lopez-Olivo [32] overall improvement was measured by a clinician or physiotherapist assessment, other studies used self reported scores [30], a single item [27] or the OARSI/OMERACT responder criteria [34] to assess overall improvement. Two studies [34,35] showed a significant but small association between outcome expectations and overall improvement on the long term, whilst on the other timepoints no associations were shown. The number of participants in these studies ranged from 51–1327 and scores on the quality assessment were between 50 and 79%.

#### Pain

Pain was used as an outcome in 9 out of 18 studies. Most studies used the WOMAC pain subscale to measure pain. No association was found between self efficacy expectations and pain at the short term (unadjusted, 1 study, N=100, quality score=53%). At the medium term (>6 weeks - ≤6 months) in the adjusted analysis both studies showing a positive and no association were found (3 studies, N=78-272, quality score = 58-74%). No study investigated the relationship between self efficacy expectations and pain after six months (long term).

The association between outcome expectations and pain was found to be unclear at the medium term in both unadjusted analysis (4 studies, N=106-2350, quality score=39-74%) and adjusted analysis (3 studies, N=78-788, quality score=58-74%). At the long term a positive association was found in the unadjusted analysis (2 studies, N=1327-2350, quality score=58-63%), however the adjusted analysis showed inconsistent results (4 studies, N=598-2350, quality score=53-74%). No study investigated the relationship between outcome expectations and pain at the short term (≤6 weeks).

#### Function

The association between patient expectations and function was assessed in 11 studies. Most frequently the WOMAC function scale was used. No association was found between self-efficacy expectations and function at the short term both in unadjusted analysis (1 study, N=100, quality score=53%) and adjusted analysis (1 study, N=100, quality score=53%). In the unadjusted analysis the same was seen for the medium term (2 studies, N=123-272, quality score =74-79%) however, in the adjusted analysis both studies showing a positive and no association were found resulting in an unclear picture (4 studies, N=78-272, quality score=58-79%). At the long term none of the studies investigated the association between self efficacy expectations and function. At the short term no association was found between outcome expectations and function (adjusted, 1 study, N=51, quality score=68%). At the medium term an unclear association was seen in both unadjusted (4 studies, N=106–788, quality score=39-74%) and adjusted (3 studies, N=222-788, quality score=58-74%) analysis. In the adjusted analysis only one out of three studies [15] found a positive association. This association however, was found when investigating the relationship between *expectations about pain* and function.

An unclear picture was also seen for the unadjusted and adjusted association between outcome expectations and function at the long term (unadjusted 4 studies, N=130-2350, quality score=6-63%) (adjusted, 4 studies, N=598-2350, quality score=52-74%).

#### Stiffness

Stiffness was assessed at the medium term by 4 studies and by one study at the long term, all used the WOMAC stiffness scale. Engel et al. [25] (N=78, quality score=58%) were the only study that addressed the relationship between self efficacy expectations and stiffness and found a positive association in the multivariable analysis, persons with a higher score on the self efficacy measure had a lower WOMAC stiffness score (β-0.260 p<0.05), indicating less stiffness. In both unadjusted (3 studies, N=106-788, quality score=39-74%) and adjusted analyses (2 studies, N=222-788, quality score=58-74%) no evidence was found for an association between outcome expectations and stiffness at the medium term. At the long term Judge [34] found a positive association between outcome expectations and stiffness at both adjusted and unadjusted analyses.

#### Satisfaction

Satisfaction was assessed at the medium term in 4 studies [24,26,27,31] and at the long term in 1 study [35], in three of these as only outcome of interest. The measurement of both the pre-operative expectation variable and the satisfaction variable differed highly amongst the studies. On the medium term three studies concluded that outcome expectations did not influence satisfaction (N=44-598, quality score= 50-61%). Mannion [27] (N=146, quality score=58%) however found that both outcome expectations about post-operative pain and outcome expectations about functional recovery were correlated with satisfaction, though these expectation items did not make a unique significant contribution to explaining the variance in satisfaction in the multiple regression analysis. Arden [35] found on the long term that expectations about post-operative pain were not correlated with satisfaction but that outcome expectations about functional recovery were correlated with satisfaction.

Overall, when analyses were adjusted for confounding factors, no relationship was seen between outcome expectations and satisfaction. No study examined the relationship between self-efficacy expectations and satisfaction.

## Discussion

Recently, patients’ expectations prior to surgery have gained attention as a predictor of TKA and THA outcomes. However, a systematic overview was lacking. This systematic review therefore aimed to summarize all the available evidence for a relationship between different types of patients’ expectations prior to TKA and THA and the 5 most important treatment outcomes of TKA and THA at 3 different timepoints.

The associations between self efficacy and outcome expectations and pain (medium and long term) and self efficacy and outcome expectations and function (medium term) were the ones that were most in favour of an actual relationship between expectations and outcomes. Still, within these comparisons both studies showing positive and studies showing no association were found. For the other outcomes and timepoints no substantial associations were found.

The results of this study have to be interpreted in the light of some strengths and limitations. Firstly, we noticed that studies that included patient expectations as one of the many variables in their models were hard to find by assessing titles and abstracts of studies. Therefore we might have missed studies that included an expectation measure in their multivariable models but did not report it in their abstract because findings were non-significant. Therefore, we randomly selected 50 articles that were initially excluded based on their title and abstract and screened the methods and results sections of those articles. In this selection of articles there was no indication that we missed relevant studies.

Secondly, meta-analysis was not possible in the current review due to heterogeneity in measurement methods of expectations and outcome measures. Also, the standard of reporting was poor and the data necessary for meta-analysis were frequently not reported. Therefore we decided to summarize the evidence for an association by awarding them with a + (a clear positive association), + − (unclear association), o (no association), and – (negative association) and also report the score on the methodological quality assessment and sample size in the same table. This approach has some challenges because arbitrary cut off points are used for clinically significant associations and it does not take the strength of the association into account. Meta-analysis would allow a more definite conclusion as it provides an estimate of the strength of the association, but the available data did not allow us to perform a meta-analysis.

Thirdly, the quality assessment revealed scores between 6% [37] and 79% [30]. Despite this large range in quality we decided not to exclude studies based on arbitrary cut-off points of high and low quality. The score on the quality assessment however was taken into account when drawing conclusions about the overall association. A limitation of this assessment was that it sums all positive scored items, thereby assuming all items are equally important. Moreover this procedure rates reporting of the items. In the current review for instance most studies described well how they measured expectations and therefore scored positive on this item (item 7). However, a good description of a measurement instrument not necessarily equals validity of the measurement instrument.

Important issues that need attention in future research are the theoretical framing of the patient expectations construct, the measurement of patient expectations and the correlation of expectations with other psychological factors.

First of all, we found that there are inconsistencies in definitions and terminology as well as in classification of patient expectations. This might be because the construct of expectations is not strictly defined yet and no consensus exists in scientific literature. We think that patient expectations are a multifaceted and complex construct, which is recognized in several reviews [20,38,39], Kravitz [39] for example distinguishes probability (i.e. the estimated probability that an event will occur) and value expectations (i.e. idealized expectations, hopes/wishes) whilst Bandura [18] distinguishes self efficacy expectations and outcome expectations. Recently Hobbs et al. [40] made a promising effort to classify patient’s expectations for THA by using the ICF-framework. Using such a framework can amongst other things lead to more uniformity of definitions and terminology and better validation of measurement methods. In the current review we found 9 studies [14,16,17,24,29,31,34,35,37] that did not define expectations and the other 9 studies all used different definitions. We chose to use the work by Crow et al. [20] and Bandura et al. [18,19] to classify the types of expectations included in the original studies because they extensively reviewed the literature and made an attempt to summarize the most common classifications and definitions. Still, these different perspectives on expectations make it challenging to compare and summarize effects of expectations on treatment outcomes.

Secondly, we believe that, possibly resulting from a lack of theoretical framing of the construct, the patient expectation field is plagued by poor measurement. This can have considerable influence on the results of studies and therefore also on the results of the systematic reviews. Constantino et al. [13] concluded in their review on the relationship between expectations and outcomes in psychotherapy that 67% of the measurements included were of poor quality. Van Hartingsveld [41] tried to identify and assess the clinimetric properties of published patient expectation measurement instruments in the musculoskeletal health field and concluded that ranking the measurement instruments was impossible due to heterogeneity and incomplete clinimetric data. Another issue that arises is the timing of the expectation measurement. In the current review the measurement moment of expectations ranges from six weeks until one day before surgery. As the amount of information provided and the interaction with the practitioner/physician seems to have substantial influence on expectations, it is arguable that the later expectations are measured, the more realistic they are and therefore will be stronger associated with outcomes. As far as we know no study has yet investigated which time point is best to measure expectations.

Finally, psychological factors such as catastrophizing, depression or optimism may influence treatment outcomes related to TKA/THA [32,42] or interact with expectations [43]. Still only a minority of studies included in this review adjusted for psychological factors in their analysis. Future studies should identify and consider these psychological factors in order to conclude whether the association between expectations and outcomes in TKA/THA provides unique information independent from these psychological factors or is influenced by these factors.

## Conclusion

The results of this review show that in general there is limited evidence for an association between patients expectations and treatment outcomes in TKA and THA. Moreover, this review highlights the need for more research in the field of patient expectations for TKA/THA.

## Abbreviations

TKA: Total knee arthroplasty; THA: Total hip arthroplasty; WOMAC: Western Ontario and McMaster Universities Arthritis Index; VAS: Visual Analogue Scale; PT: Physiotherapist; KOOS: Knee injury and Osteoarthritis Outcome Score; FFbH-OA: Functional Questionnaire of Hannover for Osteoarthritis; KSRS: Knee society rating system.

## Competing interests

The authors declare that they have no competing interests.

## Authors’ contributions

TMH participated in the design of the study, carried out the literature search, selection and evaluation of articles and writing of the review. EPJ carried out the literature search. TB participated in selection and evaluation of articles and revising the manuscript. RWO participated in the design of the study, selection and evaluation of articles and revising the manuscript. RWP participated in the design of the study and revising the manuscript. PC participated in the design of the study and revising the manuscript. HCWV participated in the design of the study and revising the manuscript. All authors read and approved the final manuscript.

## Supplementary Material

Additional file 1 Search strategy in PubMed. Click here for file
