# Correction: systematic review: do patient expectations influence treatment outcomes in total knee and total hip arthroplasty?

**DOI:** 10.1186/1477-7525-11-72

**Published:** 2013-04-29

**Authors:** Tsjitske M Haanstra, Tobias van den Berg, Raymond W Ostelo, Rudolf W Poolman, Elise P Jansma, Pim Cuijpers, Henrica C W de Vet

**Affiliations:** 1 Department of Epidemiology and Biostatistics and the EMGO Institute for Health and Care Research, VU University Medical Centre Amsterdam, Van der Boechorststraat 7, Amsterdam, 1081 BT, the Netherlands

## Correction

After publication of this article, it came to our attention that an author’s name was misspelled. The correct name is Elise P Jansma.

The authors and Publisher apologize to the readers for the inconvenience caused.
