# Supplementary material for: Systematic review: Do patient expectations influence treatment outcomes in total knee and total hip arthroplasty?
Source: Health Qual Life Outcomes. 2012 Dec 18;10:152. doi: 10.1186/1477-7525-10-152 (PMC3568025; doi:10.1186/1477-7525-10-152)
Supplement: Additional file 1 — Search strategy in PubMed. [file 1477-7525-10-152-S1.docx]

Additional file 1 . Search strategy in PubMed.

| **Set** | **Search terms** |
| --- | --- |
| #1 | "Arthroplasty, Replacement, Knee"[Mesh] OR "Arthroplasty, Replacement, Hip"[Mesh] OR (("Arthroplasty"[Mesh:NoExp] OR "Arthroplasty, Replacement"[Mesh:NoExp]) AND ("Knee"[Mesh] OR "Knee Joint"[Mesh] OR "Hip"[Mesh] OR "Hip Joint"[Mesh])) OR "Osteoarthritis, Knee/surgery"[Mesh:NoExp] OR "Osteoarthritis, Knee/therapy"[Mesh:NoExp] OR "Osteoarthritis, Hip/surgery"[Mesh:NoExp] OR "Osteoarthritis, Hip/therapy"[Mesh:NoExp] OR "Hip Prosthesis"[Mesh] OR "Knee Prosthesis"[Mesh] OR ("Knee Arthroplasty"[tiab] OR "knee replacement"[tiab] "hip arthroplasty"[tiab] OR "hip replacement"[tiab] OR "joint arthroplasty"[tiab] OR "knee osteoarthritis"[tiab] OR "hip osteoarthritis"[tiab] OR "knee prosthesis"[tiab] OR "hip prosthesis"[tiab] NOT medline[sb]) |
| #2 | "Self Efficacy"[Mesh] OR "Health Knowledge, Attitudes, Practice"[Mesh] OR expectation[tiab] OR expectations[tiab] OR expectancy[tiab] OR expectance[tiab] OR credibility[tiab] OR "patient preference"[tiab] OR "self efficacy" [tiab] OR "health knowledge"[tiab] OR "health attitude"[tiab] OR "attitude to health"[tiab] OR belief[tiab] |
| #3 | #1 AND #2 |
| #4 | #3 NOT ("addresses"[Publication Type] OR "biography"[Publication Type] OR "case reports"[Publication Type] OR "comment"[Publication Type] OR "directory"[Publication Type] OR "editorial"[Publication Type] OR "festschrift"[Publication Type] OR "interview"[Publication Type] OR "lectures"[Publication Type] OR "legal cases"[Publication Type] OR "legislation"[Publication Type] OR "letter"[Publication Type] OR "news"[Publication Type] OR "newspaper article"[Publication Type] OR "patient education handout"[Publication Type] OR "popular works"[Publication Type] OR "congresses"[Publication Type] OR "consensus development conference"[Publication Type] OR "consensus development conference, nih"[Publication Type] OR "practice guideline"[Publication Type]) NOT ("animals"[MeSH Terms] NOT "humans"[MeSH Terms]) |
